# Multiple copy number variation in a patient with Kleefstra syndrome

**DOI:** 10.1590/1984-0462/2024/42/2022230

**Published:** 2023-09-15

**Authors:** Thomas Nohama Lee, Henrique El Laden Rechetello, João Batista De Arêa Lima, João Pedro Fagoti Ferraz Cornelio, Naiara Bozza Pegoraro, Salmo Raskin, Liya Regina Mikami

**Affiliations:** a Faculdade Evangélica Mackenzie do Paraná, Curitiba, PR, Brazil.; b Universidade Positivo, Curitiba, PR, Brazil.; c Genetika – Centro de Aconselhamento e Laboratório de Genética, Curitiba, PR, Brazil.

**Keywords:** Neurodevelopmental disorders, Genetics, Genes, Transtornos do neurodesenvolvimento, Genética, Genes

## Abstract

**Objective:**

To report a rare case of a patient with a molecular diagnosis of Kleefstra syndrome (KS) who has four other chromosomal alterations involving pathogenic variants.

**Case description:**

Male patient, two years old, with global delay, including in neuropsychomotor development, ocular hypertelorism, broad forehead, brachycephaly, hypotonia, ligament laxity, unilateral single palmar crease and arachnoid cyst. The microarray-based comparative genomic hybridization (a-CGH) identified copy number variations (CNVs) in five regions: 9q34.3, 6p22.1, Yq11.223, Yp11.23, and 2q24.1. The heterozygous microdeletion in 9q34.3 involving the *EHMT1* gene confirms the diagnosis of KS.

**Comments:**

The presence of pathogenic CNVs and/or those of uncertain significance, located on chromosomes 2, 6 and Y, may be contributing to a variability in the patient's clinical condition (arachnoid cyst, single palmar fold and ligament laxity), compared to other individuals with only KS genetic alteration, making the dignosis of the disease harder.

## RESUMO

**Objetivo:**

Relatar um caso raro de paciente com diagnóstico molecular de síndrome de Kleefstra (SK) que apresenta quatro outras alterações cromossômicas envolvendo variantes patogênicas.

**Descrição do caso:**

Paciente masculino, dois anos de idade, com atraso global de desenvolvimento neuropsicomotor, hipertelorismo ocular, fronte ampla, braquicefalia, hipotonia, frouxidão ligamentar, prega palmar única unilateral e cisto aracnoide. Exame de hibridização genômica comparativa (a-CGH) identificou variações de número de cópias (CNV) em cinco regiões: 9q34.3, 6p22.1, Yq11.223, Yp11.23 e 2q24.1. A microdeleção heterozigótica em 9q34.3 confirma o diagnóstico de síndrome de Kleefstra.

**Comentários:**

A presença das CNV patogênicas e/ou de significado incerto, localizadas nos cromossomos 2, 6 e Y, pode estar contribuindo para uma variabilidade no quadro clínico do paciente (cisto aracnoide, prega palmar única e frouxidão ligamentar) em relação a outros indivíduos somente com alteração genética da SK, dificultando o diagnóstico da doença.

## INTRODUCTION

Kleefstra syndrome is a lesser-known and poorly described disease. Previously called 9q subtelomeric deletion syndrome, it was recognized by its name in 2010 in honor of Dr. Tjitske Kleefstra.^1^ The syndrome is mainly caused by a heterozygous microdeletion at 9q34.3 involving the *EHMT1* gene or by pathogenic variations, with almost all reported cases presenting *de novo* mutations.^2^ The *EHMT1* gene encodes the histone methyltransferase enzyme, which modifies histones (proteins that bind to the DNA, shaping the chromosome by attaching a methyl molecule to the histones). This enzyme can suppress the activity of genes responsive to the MYC and E2F proteins, a mechanism essential for proper development and normal organ function. The lack of this enzyme or defects in its functionality impairs the expression of these genes, resulting in characteristics of the syndrome.^3,4^

Some of the clinical changes observed in the patients include neurological deficit, autistic patterns, delayed speech development, childhood hypotonia, and distinctive facial features such as brachi/microcephaly, enlarged forehead, unusual eyebrow shape (curved or straight with synophrys), slightly upward leaning palpebral fissures, retrusion of the medial third of the face, thickened ear helix, short nose with anteverted nostrils, eversion of the lower lip, exaggerated labial cupid's bow or outward-facing upper lips, protruding tongue, and relative prognathism. Heart, kidney, urological, and genital defects (in men), severe respiratory infections, epilepsy, febrile convulsions, extreme apathy, and post-puberty catatonia may also be associated with the syndrome.^2^

The prevalence of KS is estimated at 1/200,000,^5^ with only 85 cases documented worldwide from 1990 to 2011.^1^ Due to the similarity of clinical and behavioral characteristics, it can be misdiagnosed as autism, especially in early childhood. When in doubt, the differential diagnosis should be made by molecular techniques, such as microarray comparative genomic hybridization (a-CGH), single gene test, multigene test panel, and, more rarely, karyotype analysis.^2^

The relevance of this study is due to the fact that it is a rare case of a patient with KS associated with multiple pathogenic copy number variations (CNVs), which possibly exacerbate the patient's clinical condition. There are no case reports in the literature similar to the one described, with multiple associated pathogenic CNVs. This paper might help the medical field by providing data regarding the disease and its aggravating features, allowing a clearer comprehension and approach, as well as clarifying how the presence of other CNVs can interfere with the clinical condition.

The objective of the present work was to report a rare case of a patient with a molecular diagnosis of Kleefstra syndrome and presenting four other chromosomal alterations — CNVs — with pathogenic variants associated with chromosomes 2, 6, and Y.

## CASE REPORT

This study was approved by the Ethics Committee of the Faculdade Evangélica Mackenzie do Paraná (FEMPAR), under opinion no. 241.525.

A retrospective study was conducted by analyzing the patient's medical records. The following data were collected: gender, age, pedigree with a family history of genetic diseases and congenital anomalies, history of neuropsychomotor development, congenital physical alterations, genetic tests, and other laboratory and imaging tests performed. To maintain the patient's and his family's privacy, any identifying information was kept confidential and coded.

The a-CGH test consisted of oligonucleotide-based comparative genomic hybridization using DNA microarrays. These are solid surfaces on which nucleic acids are immobilized and used as targets for hybridization, which can be performed by a-CGH. This technique essentially consists of chromosomal analysis by microarray, detecting duplications or deletions of the genetic material, in comparison with normal DNA.^6^

Genetic analysis was performed with the GRCh37/hg19 version of the genome. The platform used in this examination was CytoSNP 850K, which contains about 850,000 oligonucleotides distributed throughout the human genome. CNVs larger than 50 Kb have been described. The single nucleotide polymorphism (SNP) component of this array allows analysis of the absence of heterozygosity (AOH). The results were interpreted using the Database of Genomic Variants (DGV), Online Mendelian Inheritance in Man (OMIM), DECIPHER and additional available databases. Copy number variant classification is based on the American College of Medical Genetics and Genomics standards and guidelines for interpretation of postnatal constitutional copy number variants. Copy number variants are evaluated based on the reason for referring the patient for this genomic test. Classification of variants according to the American College of Medical Genetics and Genomics:

1. Pathogenic (class 1) — variant with sufficient evidence to confirm pathogenicity;
2. Probably pathogenic (class 2) — variant with strong evidence in favor of pathogenicity;
3. Uncertain significance (class 3) — variant with limited and/or conflicting evidence for pathogenicity;
4. Probably benign (class 4) — variant with strong evidence of benignity (or non-pathogenicity);
5. Benign (class 5) — variant with sufficient evidence to confirm no pathogenicity, also known as polymorphisms.

A 2-year-old male patient, with non-consanguineous parents, no family history of genetic diseases or congenital anomalies, presented with global neuropsychomotor developmental delay, delayed speech development (beginning at one year and four months), ocular hypertelorism, wide forehead, brachycephaly, hypotonia, ligamentous laxity, arachnoid cyst and unilateral single palmar crease, which led to the diagnostic hypothesis of autism spectrum disorder (ASD).

Regarding his development, he held his head up at three months, sat independently at eight months, did not crawl, walked at two years and six months, and began to speak in complete sentences at four years. No specific tests were performed for the diagnosis of ASD, only the neuropediatric clinical exam. In 2023, he is a nine-year-old boy in the 4^th^ year of elementary school, studying at a regular school. He does not have intellectual disability but has learning difficulties. Regarding social interaction, he can play and interact with children of the same age.

Karyotype and X-fragile tests showed no chromosomal abnormalities. Brain magnetic resonance imaging (MRI) was normal and spinal MRI showed a spinal cyst. Due to the results presented, a-CGH was indicated to verify submicroscopic chromosomal alterations.

This test identified five CNVs: a pathogenic heterozygous microdeletion of 54,411 Kb on 9q34.3, partially involving the *EHMT1* gene compatible with the diagnosis of Kleefstra syndrome; a heterozygous microdeletion of uncertain significance, never described in the literature, of 64,229 Kb on 6p22.1, involving the *OR2J2* gene; a heterozygous microduplication, also of uncertain significance, of 301,254 Kb on 2q24.1 involving the TANC 1 and DAPL1 genes; and two pathogenic heterozygous microdeletions on the Y chromosome, one of 1,731,976 Kb on Yq11.223 and another of 116,811 Kb on Yp11.23, involving the *PRY2, PRY, TTTY6, TTTY5, TTTY17A, TTTY4, DAZ1, DAZ2* and *DAZ3*

## Discussion

The alteration identified in 9q34.3 is consistent with the diagnosis of Kleefstra syndrome. Regarding the other pathogenic alterations, in the DECIPHER database, which presents unpublished clinical data of individuals with genetic alterations, there are reports of patients with pathogenic, or likely pathogenic, alterations similar to those found in this case, but not of the same size and involving the same genes.^7^

Microdeletions in 6p22.1, involving the *OR2J2* gene, have been reported in patients with neurological deficits, autistic features, delayed speech development, hypotonia in childhood, and distinctive facial characteristics. Chen et al., in a study on single nucleotide polymorphism, evidenced the correlation between the 6p22.1 region with cases of schizophrenia and decreased gray matter volume.^8^

**Figure 1:**
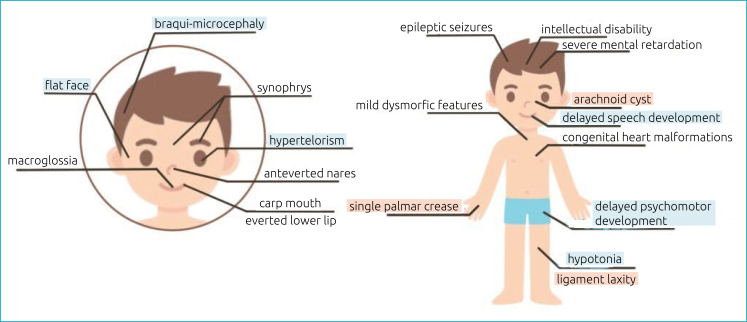
Clinical features of Kleefstra syndrome. Kleefstra syndrome-related features found in the patient highlighted in blue. Features unrelated to Kleefstra syndrome found in the patient highlighted in red.

In the DECIPHER database, microdeletions in Yq11.223, involving the *PRY*, *PRY2*, *TTTY5,* and *TTTY6* genes, were found in a patient with delayed speech development and mild global developmental delay. Such alterations resemble the clinical features of the patient in this study; however, it cannot be said that they are due to this alteration, since these are also clinical characteristics found in Kleesftra syndrome (Figure 1). Studies indicate a possible link between the Yq11.223 region and spermatogenesis since the *PRY* and *PRY2* genes were often deleted in infertile male patients.^9,10^ Wong et al. demonstrated the presence of *PRY* mRNA in the testis, as well as in the brain and skeletal muscle.^11^ As the patient in this study has not yet reached fertile age it is not possible to establish links between defects in the spermatogenesis process and his clinical case. There is a scarcity of studies on CNVs in certain genes of the Yq11.223 region, complicating the correlation between the mutations and the clinical findings of the patient.

Microduplication at 2q24.1 involving *TANC1* and *DAPL1* genes has been reported in a patient in the DECIPHER database who presented the following features: interatrial septal defect, intestinal malrotation, bilateral ptosis, intrauterine growth restriction, camptodactyly, neonatal hypotonia, severe global developmental delay, profound intellectual development, and seizures. *TANCs* genes have been related to the modulation of neuron projection and synapse plasticity.^12^ Han et al., in a study *in vitro* and in mice, demonstrated that this gene has a function in regulating the density of dendritic spines and excitatory synapses.^13^ This alteration would cause an increase in the expression of the *TANC1* gene, consequently increasing the density of dendritic spines. Hustler and Zhang demonstrated the correlation between a high density of dendritic spines with lower brain weights, being more commonly found in ASD patients with lower levels of cognitive function.^14^

In contrast, reports of similar alterations in Yq11.23 showed no clinical correlation with the patient. Deletions in this region have been related to azoospermia, and consequently to male fertility problems.^15^ These findings do not corroborate the patient's clinical case of the current study.

It can be inferred that the presence of other pathogenic CNVs, located on chromosomes 2, 6, and Y, may be contributing to the phenotypic variability observed in the patient's clinical picture in relation to other individuals with only submicroscopic chromosomal alteration of KS, since the presence of arachnoid cyst, ligament laxity and single palmar crease are not features commonly found in patients with Kleefstra syndrome (Figure 1). However, since variants of uncertain significance on chromosomes 2 and 6 have never been described in the literature and variants on the Y chromosome have been correlated with the clinic only in adult males, it cannot be stated that they are directly associated with the three atypical clinical findings of the patient.

Corroborating this finding, no other case of a patient with this syndrome associated with four other chromosomal abnormalities in the same individual, such as those presented by the reported patient, was found in the literature. In the DECIPHER database, 32 cases of pathogenic microdeletions in *EHMT1* were reported, of which only six showed an association with other CNVs. In only one patient was the additional CNV considered pathogenic. Other four patients had likely pathogenic CNVs. The highest number of associated CNVs found in a case was three, presenting two benign CNVs and one of uncertain significance on a patient with hypertelorism, small mouth, microcephaly, anteriorized anus, and defects in the interatrial and interventricular septa, characteristics completely different from those presented by the patient presented in this study.

In conclusion, the a-CGH result confirms Kleefstra syndrome, but associated with multiple CNVs. The syndrome is known to be related to neuropsychomotor development deficits. The presence of other CNVs in regions related to nervous system development, on chromosomes 2, 6, and Y, corroborates the phenotypic variability in the clinical picture presented by the patient, differentiating him from a typical case. The a-CGH test was essential for the diagnosis of the disease, since it excluded the initial diagnosis of ASD and provided an early diagnosis and an accurate prognosis of the patient's disease, allowing a better understanding of his peculiar clinical condition and possible future complications.
